# Uterotonic Drugs in Prevention and Management in Postpartum Haemorrhage in Prehospital Deliveries—A Systematic Review

**DOI:** 10.3390/healthcare13090976

**Published:** 2025-04-23

**Authors:** Hanna Wiciak, Mateusz Strózik, Jacek Smereka

**Affiliations:** 1 Clinical Department of Gynecologic Surgery and Oncology, University Center of Obstetrics and Gynecology, Wroclaw Medical University, 50-367 Wroclaw, Poland; hanna.wiciak@umw.edu.pl; 2 Clinical Department of Obstetrics and Gynecology, University Center of Obstetrics and Gynecology, Wroclaw Medical University, 50-367 Wroclaw, Poland; m.strozik@umw.edu.pl; 3 Department of Emergency Medical Service, Wroclaw Medical University, 50-367 Wroclaw, Poland

**Keywords:** ambulance, paramedic, prehospital delivery, postpartum haemorrhage, oxytocin, uterotonic drugs

## Abstract

Background: Obstetric haemorrhage, particularly postpartum haemorrhage (PPH), remains a significant global health challenge and a leading cause of maternal mortality. Despite advancements in understanding and preventing PPH, haemorrhage remains a leading cause of maternal mortality worldwide. The aim of this study was to review the current literature on the use of uterotonic drugs, particularly oxytocin, in reducing perinatal mortality during prehospital deliveries. Methods: In December 2024, a comprehensive search was conducted across PubMed, Web of Science, Embase, and Scopus, yielding 108 records, of which four studies met the inclusion criteria. Results: The limited evidence underscores the need for targeted research and adherence to international obstetric guidelines to improve PPH management and maternal outcomes. In some countries, the only uterotonic drug available in all EMS teams is oxytocin; in others, there is none. Emergency Medical Services (EMS) play a critical role in providing lifesaving interventions during obstetric emergencies, often serving as the first and sometimes only point of medical contact for women experiencing complications during childbirth. Conclusion: There is a lack of high-quality clinical studies evaluating the effectiveness of uterotonic agents in EMS operations and their role in treating postpartum haemorrhage in prehospital settings. Addressing this gap requires targeted research to generate robust evidence and inform the development of standardized protocols. Such efforts could enhance the timely management of PPH, ultimately reducing maternal mortality and improving outcomes in resource-limited and prehospital environments. By bridging the evidence gap, EMS systems worldwide can be better equipped to handle obstetric emergencies effectively.

## 1. Introduction

Obstetric haemorrhage, particularly postpartum haemorrhage (PPH), remains a significant global health challenge and a leading cause of maternal mortality. Despite advancements in understanding and preventing PPH, haemorrhage remains a leading cause of maternal mortality worldwide. According to a systematic analysis, haemorrhage was responsible for 34% of the 275,000 estimated global maternal deaths in 2015, highlighting the critical need for sustained efforts to improve prevention, management, and access to effective interventions, particularly in resource-limited settings [1].

Although the definitions of postpartum haemorrhage vary among scientific organizations—the World Health Organization (WHO) [2] defines it as blood loss of >500 mL within 24 h of childbirth, the American College of Obstetricians and Gynecologists (ACOG) [3] as >1000 mL within the same timeframe, and the Society of Obstetricians and Gynaecologists (SOGC) [4] as any blood loss that threatens haemodynamic stability—research published to date indicates that the uterotonic is the most important component in preventing PPH [5]. Despite these definitional differences, research consistently identifies uterotonic agents—especially oxytocin—as the cornerstone of PPH prevention.

While previous systematic reviews, such as De Silva’s, have explored PPH management in hospital settings, limited attention has been given to prehospital care, where Emergency Medical Services (EMS) often provide the first and sometimes only medical intervention [6]. Given the variability in EMS protocols and the availability of uterotonic drugs, a focused review of their role in prehospital settings is necessary. This study aims to evaluate the existing literature on the use of uterotonic drugs by EMS in managing PPH, identifying gaps in knowledge and areas for improvement.

## 2. Material and Methods

### 2.1. Study Design

This systematic review was conducted in adherence to the PRISMA (Preferred Reporting Items for Systematic Reviews and Meta-Analyses) guidelines to ensure methodological transparency and consistency in the analysis process. The PICO framework (Population, Intervention, Comparison, Outcome) was used to define the research question and guide the selection process.

### 2.2. Research Question

Using the PICO model, the research question was defined as follows: What is the impact of uterotonic drug administration in a prehospital setting on perinatal outcomes, particularly in the context of deliveries managed by Emergency Medical Services (EMS)?

- Population (P): Women giving birth in prehospital settings, specifically deliveries occurring outside hospital facilities and attended by emergency medical services (EMS) teams.
- Intervention (I): Administration of uterotonic drugs (e.g., oxytocin, carbetocin, misoprostol, ergometrine) to prevent or manage obstetric complications such as postpartum haemorrhage.
- Comparison (C): No uterotonic treatment or alternative management strategies.
- Outcome (O): Perinatal outcomes (including maternal and neonatal mortality) and other clinical outcomes, such as postpartum haemorrhage, neonatal complications, and adverse events related to uterotonic use.

The PICO framework guided the search strategy development and the selection criteria for eligible studies, ensuring that the review systematically addressed the impact of uterotonic drugs in prehospital obstetric care.

### 2.3. Search Strategy

A comprehensive database search was concluded in December 2024 using four electronic databases—PubMed, Web of Science, Embase, and Scopus—to identify articles addressing the central research question. The search strategy involved combining terms related to the following:

- Uterotonic agents: “uterotonic drugs”, “oxytocin”, “misoprostol”, “ergometrine”, and “carbetocin”;
- Prehospital obstetric care: “prehospital delivery”, “out-of-hospital birth”, “emergency obstetric care”, “paramedics”, “ambulance”, and “EMS obstetric care”;
- Outcomes: “perinatal mortality”, “maternal mortality”, and “neonatal outcomes”.

Search terms were adjusted for each database’s indexing system. No publication date limits were applied. Only articles published in English were included.

### 2.4. Eligibility Criteria

Inclusion Criteria:

- Studies involving the administration of uterotonic agents in prehospital childbirth attended by EMS;
- Clinical trials, observational studies, or systemic reviews with extractable data;
- Studies reporting on maternal or neonatal outcomes.

Exclusion criteria:

- Studies conducted exclusively in hospital settings;
- Case reports, editorials, opinion pieces, and letters without original data;
- Articles not available in full-text or not in English.

Rationale:

Due to the limited number of studies available on prehospital PPH management, broad inclusion criteria were used to maximize the scope of evidence while maintaining relevance to the research question.

### 2.5. Study Selection

A total of 108 articles were identified across all databases. After eliminating 41 duplicates, 67 unique articles remained. Titles and abstracts were screened for relevance by two independent reviewers. Full-text review was conducted for eligible articles, resulting in 4 studies included in the final analysis.

### 2.6. Data Extraction

Data were extracted independently by two reviewers into a structured table capturing the following:

- Study design and setting;
- Population characteristics;
- Type of uterotonic used;
- EMS protocols or procedures;
- Reported outcomes (e.g., maternal mortality, PPH rates, adverse events).

Disagreements were resolved through discussion.

### 2.7. Quality Assessment

Due to the small number of included studies, a formal risk of bias assessment was not feasible. However, methodological quality was considered during narrative synthesis, and limitations were acknowledged in the discussion.

### 2.8. Data Synthesis

Given the heterogeneity in study design, outcome reporting, and intervention specifics, a narrative synthesis approach was adopted instead of a meta-analysis. Key findings were summarized descriptively and compared thematically.

### 2.9. Flow Diagram

**Figure 1:**
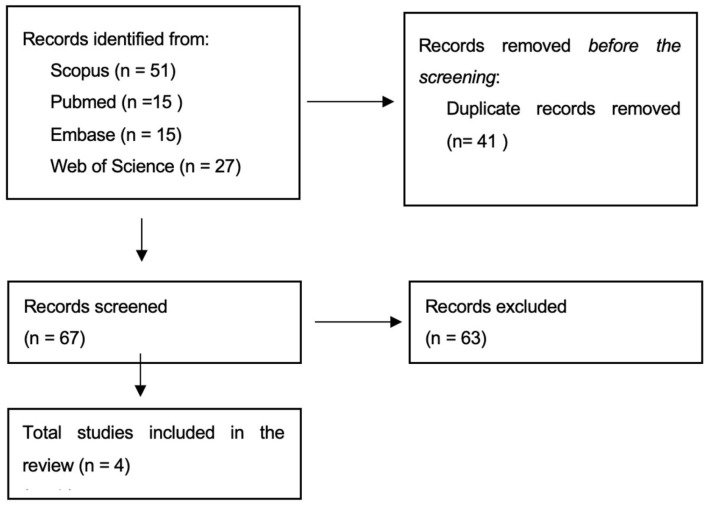
PRISMA flow diagram showing study selection.

A Reporting Items for Systematic Review and Meta-analysis (PRISMA) flow diagram was utilized to visually present the study selection process (Figure 1).

## 3. Results

### 3.1. Study Characteristics

A total of four studies met the inclusion criteria for this review. These included one systematic review and three retrospective observational studies published between 2020 and 2024. Sample sizes ranged from 62 to 350 participants or cases. All studies focused on using oxytocin in prehospital or out-of-hospital (OOH) obstetric care provided by Emergency Medical Services (EMS). The included studies were conducted in different geographical regions and varied in terms of protocols, population, and specific outcomes assessed (Table 1).

### 3.2. Interventions and Outcomes

Across all studies, oxytocin was the main uterotonic agent investigated. The systematic review by De Silva et al. compared oxytocin with tranexamic acid (TXA) and concluded that in the out-of-hospital environment, there were no significant differences in blood loss, maternal or neonatal morbidity, or need for further interventions between oxytocin and standard care. This study represented the most comprehensive approach but included a broader definition of “out-of-hospital”, encompassing rural and low-resource environments [6].

The observational studies focused primarily on oxytocin’s real-world use and outcomes in EMS contexts. Wiciak et al. analysed EMS records from Poland and found that paramedics rarely administrated oxytocin despite its inclusion in national protocols [7]. Schultz et al. described oxytocin as a well-accepted and safe addition in management during the third stage of labour, suggesting that the EMS system should consider using it as a routine [8]. However, Klemettilä et al. reported no significant association between oxytocin use and a reduction in PPH, raising questions about its clinical effectiveness when administered late or under uncontrolled prehospital conditions [9].

### 3.3. Quality Assessment

The Newcastle–Ottawa Scale (NOS) guided the evaluation of the methodological quality of the included studies. The systematic review by De Silva scored 8 out of 9 points, reflecting a strong methodological design, robust inclusion criteria, and use of comparative groups. The three observational studies (Klemettilä, Schultz, Wiciak) [7,8,9] scored between 5 and 6 points, mainly due to limitations such as the absence of randomization, control groups, and follow-up data. Despite these limitations, they provided significant contextual evidence regarding the feasibility and safety of uterotonic use in EMS practice.

### 3.4. Comparison with Existing Literature

The discrepancy between the number of studies included in this review (*n* = 4) and those reported in De Silva’s review (*n* = 6) can be attributed to differences in inclusion criteria and review focus. While De Silva et al. examined a broader range of interventions (e.g., including TXA) and settings (including rural, but not strictly EMS-attended births), the present review applied narrower eligibility criteria emphasizing prehospital obstetric care conducted by EMS personnel and specifically the administration of uterotonics like oxytocin [6]. This narrower scope strengthens the specificity and relevance of the findings to EMS systems but also highlights the limited volume of high-quality research available on this topic.

## 4. Discussion

The management of postpartum haemorrhage (PPH) in out-of-hospital deliveries remains a unique challenge for emergency medical services (EMS) worldwide. Our review points out critical gaps in prehospital care, including variability in uterotonic drug use, limited EMS protocols, and significant logistical barriers. While oxytocin continues to be a cornerstone of PPH prevention in clinical settings, its role in the emergency context is still uncertain and underexplored.

### 4.1. Prehospital Deliveries

Prehospital birth refers to a situation known as birth before arrival or an unplanned out-of-hospital delivery during which a newborn is delivered unexpectedly outside of a hospital setting. This is a highly challenging and exceptional situation, as these events occur suddenly in places that lack proper equipment and often do not have adequately trained or experienced personnel on-site [10]. Out-of-hospital (OOH) emergency deliveries are often viewed by emergency medical service (EMS) providers as either a uniquely joyful experience or one of the most daunting and challenging moments of their careers [11,12].

Prehospital deliveries are associated with certain risks of complications and unpredictable courses resulting from various factors. First and foremost, the issue of proper qualification for a home birth conducted in an elective setting, often with the participation of a midwife, is important. In some countries, the percentage of these births is relatively high. In the Netherlands, the percentage of home births was 16.3% in both 2015 and 2019. In comparison, other countries reported much lower rates: 1.4% in Denmark, 1.3% in Germany, 1.1% in Belgium, 0.9% in Hungary, 0.32% in Spain, 0.2% in Finland, and 0.03% in Poland [13]. Another issue is that emergency deliveries are not initially planned as home deliveries, including deliveries attended by EMS team personnel [14]. The EMS teams’ staff, physicians, nurses, and paramedics are properly trained to assist patients during out-of-hospital deliveries, including treating complications associated with childbirth and caring for the newborn [15]. There are significant gaps in the training of EMS teams, particularly in managing postpartum haemorrhage. Additionally, there are issues with the documentation process, which is often incomplete and lacks important details, including an assessment of the severity of PPH [16].

A critical issue is the ability of EMS teams to assist with postpartum haemorrhage, including the availability of appropriate medications in the ambulance and transparent procedures for their use [17]. In many countries, oxytocin and other similarly acting drugs are not standard in EMS ambulances, while paramedics are not allowed to administer the drug without a physician’s order [8,18].

Another crucial aspect of unplanned out-of-hospital delivery is the poorer outcomes for the newborn. Currently, there is no doubt that these deliveries are associated with an increased risk of perinatal mortality and neonatal morbidity, regardless of the gestational age. Several studies highlight the important role of the EMS call handler in providing essential guidance during prehospital deliveries. Key themes identified in the call-handler advice include the following: (1) the importance of neonatal temperature, (2) where to place the baby after birth, (3) methods to keep the baby warm, (4) the timing of temperature management, and (5) the clarity and priority of instructions. Proper training of EMS teams can significantly improve perinatal outcomes in prehospital settings [19,20,21,22].

From the perspective of emergency medical care, it is essential to note that prolonged transportation time is a significant predictor of neonatal mortality. Additionally, neonatal interventions before and during transport significantly impact neonatal morbidity and mortality [23,24,25].

Since paramedics most often assist BBA patients, adequate training (including practical skills) is essential to effectively care for them during this period. Paramedics must build confidence in their ability to support the newborn’s adjustment to the extra-uterine environment and ensure that the third stage of labour progresses without complications for the mother. Furthermore, ideally, they should possess the clinical skills to respond promptly to any complications that may arise.

An issue of importance is the potential cooperation of paramedics and midwives at the prehospital stage, including, in particular, the education of paramedics in assisting with out-of-hospital deliveries [26].

### 4.2. Novelty and Aim

To our knowledge, this is the first systematic review focused specifically on the use of uterotonic agents—particularly oxytocin—by emergency medical services (EMS) teams during out-of-hospital deliveries. While active management of the third stage of labour is widely recognized in obstetric care, its implementation in emergency and out-of-hospital settings remains underexplored.

### 4.3. Interpretation of Findings

The studies reviewed report that oxytocin administration in prehospital deliveries is inconsistent and often very restricted. Schultz et al. [8] showed relatively frequent administration of oxytocin in Australia (63.4%), while in Finland [9] and Poland [7], oxytocin use was markedly lower due to logistical and regulatory limitations. Notably, none of the included studies proved a significant decrease in the incidence or severity of postpartum haemorrhage (PPH) associated with prehospital oxytocin use. While safety was generally affirmed, the lack of a consistent benefit suggests other factors—such as timing, route of administration, and staff training—may affect outcomes.

### 4.4. International Guidelines and Recommendations

The recommendations from the main scientific organizations that issue guidelines for the management of postpartum haemorrhage, such as ACOG (American College of Obstetricians and Gynecologists), RCOG (Royal College of Obstetricians and Gynaecologists), FIGO (International Federation of Gynecology and Obstetrics), RANZCOG (Royal Australian and New Zealand College of Obstetricians and Gynaecologists), SOGC (Society of Obstetricians and Gynaecologists of Canada), and the WHO (World Health Organization), despite variations, are consistent in highlighting the importance of the active management of the third stage of labour for preventing postpartum haemorrhage. They recommend several interventions, with oxytocin being the standard criterion [27,28,29,30,31]. The European Society of Anaesthesiology and Intensive Care emphasizes the critical need for rapid intervention to prevent and manage postpartum haemorrhage. Quick action significantly improves the chances of therapeutic success. Furthermore, when possible in prehospital settings, 1 g of tranexamic acid is recommended, as it can aid in controlling the haemorrhage [32]. The collected and summarized recommendations for treating PPH are presented in Table 2. The authors did not find direct guidelines regarding EMS and perinatal care. The absence of unified, enforceable protocols in EMS systems undermines the consistent application of these evidence-based recommendations in out-of-hospital environments. The value of adopting globally endorsed protocols cannot be overstated: they provide clarity, support clinical decision-making under pressure, and ensure that all in-labour patients—regardless of setting—receive a minimum standard of care. Inconsistencies in drug availability and administration authority between regions or services risk preventable maternal morbidity and mortality.

### 4.5. Uterotonic Drugs in Prehospital Deliveries Attended by EMS Teams

After reviewing the literature, only a few articles on the use of uterotonic drugs in prehospital deliveries involving EMS personnel were found.

A study published by Schultz et al. in 2021 retrospectively analysed all out-of-hospital deliveries in 2018 attended by Queensland Ambulance Service teams in Australia. Of 350 out-of-hospital deliveries, oxytocin was administered in 222 cases (63.4%). In contrast, in 19.1% of cases, patients refused oxytocin administration, and in 17.4% of cases, the paramedic decided not to administer oxytocin. There were no cases of complications or side effects associated with the administration of oxytocin, which was, on average, administered 14 min after the birth of the child [8].

Klemettilä et al. performed a retrospective analysis of out-of-hospital deliveries in the area covered by Helsinki University Hospital in Finland over 5 years from 2013 to 2017. The authors found that oxytocin was available in ambulances serving half of the study population while not in the rest. During the study period, 216 out-of-hospital deliveries were found, with oxytocin available in ambulances in 111 cases. In half of these cases (57 of the 111 deliveries), oxytocin was administered. However, there was no difference in the severity of postpartum haemorrhage compared to cases in which oxytocin was not given. The study’s conclusions suggested that oxytocin is not essential in emergency team settings [9].

Wiciak et al. analysed cases of oxytocin administration between 2018 and 2023 in Poland. The authors showed that EMS teams used oxytocin in only 62 out-of-hospital deliveries, representing less than 7% of all 879 deliveries in this period in which EMS teams in Poland were involved. The percentage of oxytocin use is very low due to its availability in ambulances and the ability of paramedics to administer it [7].

The authors’ opinion is that the results of our systematic review are highly surprising, and contrary to the expected results. Our study revealed no significant reduction in postpartum haemorrhage despite widespread oxytocin in labour rooms. The lack of comprehensive studies, the absence of unified administration protocols, and potential delays or imprecise drug delivery in prehospital environments significantly challenge the systematic evaluation of oxytocin’s effectiveness in out-of-hospital settings.

Several factors may explain why oxytocin has not significantly reduced blood loss in the prehospital setting. Delays in drug administration due to post-delivery logistics, managing a newborn as another patient for the same team, or transport priorities may reduce its effectiveness. Medication routes of administration might play a role: intramuscular administration—more common in an out-of-hospital environment—may have a slower onset than intravenous administration.

Although EMS teams are usually equipped to monitor vital signs, continuous or comprehensive maternal monitoring—such as accurate blood loss estimation and uterine assessment—is often limited in out-of-hospital settings. This limits timely clinical actions and data collection for evaluating outcomes.

Furthermore, unlike obstetric teams in hospital settings, EMS personnel typically lack the equipment and training to perform gynaecological assessments such as per vaginam examination or speculum inspection. These skills are essential for identifying common sources of postpartum haemorrhages—such as retained placental tissue or birth canal lacerations—and their absence may delay accurate diagnosis and intervention in the prehospital setting.

To sum up, EMS personnel may lack experience, confidence, and time in diagnosing and managing early signs of PPH. Finally, incomplete documentation and a lack of real-time monitoring limit both response accuracy and data collection for evaluating outcomes.

### 4.6. Advances in PPH Prevention and Treatment Research

Efforts are ongoing worldwide to develop a method that is 100% effective, safe for patients, and easily accessible [33]. Such a solution would be applicable in hospital settings, suitable for home births, and used by emergency medical teams, ensuring broader reach and adaptability in diverse childbirth scenarios.

In low-resource settings, the effective use of oxytocin is often hindered by challenges related to manufacturing quality and degradation caused by inadequate cold chain management [34]. Heat-stable carbetocin (HSC) has emerged as a promising alternative, with multiple studies confirming its effectiveness in preventing postpartum haemorrhage. A single 100 mcg dose of HSC has been shown to have similar efficacy to 10 units of oxytocin [35].

While its effectiveness has also been demonstrated in caesarean deliveries, many experts remain cautiously optimistic. This is primarily due to concerns regarding the cost-effectiveness of HSC, particularly when considering its implementation in resource-constrained healthcare systems [36].

The development of a thermostable, easy-to-administer microneedle (MN) patch for oxytocin delivery holds significant potential for use by emergency medical teams, particularly in low-resource settings. This innovation addresses the logistical challenges of transporting and storing oxytocin, which requires refrigeration.

It is designed to administer 10 IU (16.8 μg) of oxytocin, providing an efficient and reliable prevention and treatment method for postpartum haemorrhage.

In emergencies, such as during home births or remote medical interventions, MN patches could provide a quick, reliable method for administering oxytocin, even by healthcare workers with limited training, such as traditional birth attendants or paramedics [37].

There is growing hope for the future in combination therapy. Numerous studies have shown that using two or more drugs in tandem increases the effectiveness of preventing postpartum haemorrhage. For example, combinations such as ergometrine plus oxytocin, carbetocin, and misoprostol plus oxytocin have proven more effective uterotonic strategies than the current standard, oxytocin alone. However, combinations like ergometrine plus oxytocin and misoprostol plus oxytocin are associated with significant side effects. As a result, there is potential for developing combined formulations that incorporate several drugs, aiming to maximize efficacy while minimizing adverse effects [38].

An emerging and effective therapy for postpartum haemorrhage involves the use of oxytocin in nasal or inhalable forms. This non-invasive delivery method has shown promise, particularly in emergency settings where rapid intervention is critical. Nasal spray formulations of uterotonics, currently under development, offer a quick and accessible alternative to traditional methods of administration, such as injections or intravenous access. These nasal sprays are especially advantageous for healthcare workers with limited training, including traditional birth attendants and emergency medical teams, who may be required to act quickly in challenging environments. By eliminating the need for needles, these spray formulations make it easier for non-specialist personnel to administer lifesaving drugs, particularly in remote or resource-limited settings. This approach holds great potential for improving the management of PPH in emergencies where time and access to medical facilities are limited [39,40,41].

### 4.7. Implications for Practice

Despite limited proof of efficacy in current studies, the routine inclusion of oxytocin in prehospital obstetric care may still be justified due to its established safety profile and strong endorsement by global authorities. The significant barriers are systemic rather than clinical. Broader inclusion of oxytocin in EMS protocols, expanded scope-of-practice for paramedics, and targeted education may improve consistency and potential outcomes. Furthermore, the promising development of heat-stable, easy-to-administer formulations (e.g., microneedle patches and nasal sprays) could significantly improve feasibility in low-resource and field settings.

### 4.8. Limitations

Only articles published in English were considered in the analysis. The analysed articles presented cases of the use of uterotonic drugs by emergency medical teams in prehospital care. Therefore, given the limited number of studies that met the inclusion criteria, we opted not to perform a meta-analysis, which would have quantitatively combined data from multiple studies. Instead, our findings and recommendations are based on an assessment of study quality and a synthesis of the available evidence rather than aggregated quantitative measures. The nature of these analyses limited the possibility of drawing far-reaching conclusions. The review included only four studies, all observational and geographically restricted, limiting generalizability. No randomized controlled trials were identified, and heterogeneity in research protocols and outcome reporting indicates difficulty in comparison. Additionally, variability in EMS systems and protocols across regions results in challenges in standardizing conclusions, further limiting the applicability of the findings to diverse healthcare contexts.

### 4.9. Future Research

Future studies should focus on evaluating standardized oxytocin administration protocols within EMS systems, ideally through prospective or controlled designs. Research should explore more up-to-date delivery systems suited to the settings context, such as non-injectable formulations. Crucially, EMS-focused guidelines for obstetric emergencies—including medication protocols—must be developed to align more closely with hospital-based standards.

## 5. Conclusions

While oxytocin is widely recognized as a first-line agent for preventing postpartum haemorrhage in hospital settings, its use in prehospital obstetric care remains inconsistent and under-researched. The evidence suggests no apparent reduction in PPH rates and no safety concerns when administered by EMS teams. To improve care for out-of-hospital deliveries, EMS systems should consider updating protocols to include oxytocin, invest in practical training, and advocate for the legal authority of paramedics to administer uterotonics autonomously. Future research and innovation—including novel delivery systems—are needed to close the gap between international recommendations and real-world EMS practice.

To improve care for out-of-hospital deliveries, EMS systems should include oxytocin in their protocols and align with unified international recommendations such as those from the WHO and FIGO. Applying these standardized, evidence-based protocols across prehospital systems would result in equity, enhance safety, and support frontline providers in delivering optimal care while managing obstetric emergencies. Practical training, regulatory reform, and investment in accessible uterotonic delivery options must accompany these efforts to bridge the gap between guidelines and real-world EMS practice.

## Figures and Tables

**Table 1:** Studies addressing the impact of uterotonic drugs in prehospital obstetric care.

Author, Year	Sample Size	Aim	Participant Characteristics	Study Design	Main Results	Newcastle–Ottawa Quality Score
De Silva et al., 2022 [6]	6 studies included in the review	To determine the efficacy of pharmacological management: oxytocin, compared to TXA, in women suffering PPH in the out-of-hospital environment.	Studies including women with PPH in rural or out-of-hospital settings.	Systematic review	In the out-of-hospital setting, there is no difference in blood loss, neonatal or maternal mortality, morbidity, or need for further interventions when using oxytocin compared to no intervention or standard care for PPH.	8
Wiciak et al., 2024 [7]	62 real situations when paramedics used oxytocin	To assess the rate of oxytocin use by paramedics during out-of-hospital births.	Women delivering outside of hospital settings in Poland; retrospective data from EMS services.	Retrospective observational study.	Polish EMS teams rarely administer oxytocin at the prehospital stage.	6
Schultz et al., 2021 [8]	350 OOH births were included in this study	Describes the prehospital administration of oxytocin by paramedics following attendance of out-of-hospital (OOH) births.	Retrospective review of EMS data.	Retrospective observational study.	Oxytocin is a well-accepted and safe treatment adjunct for the management of the third stage of labour in OOH births. It should be considered for routine practice by other emergency medical services.	5
E Klemettilä et al., 2020 [9]	216 analysed out-of-hospital deliveries	To determine whether the use of oxytocin is associated with diminished postpartum haemorrhage after unplanned out-of-hospital deliveries.	Women with unplanned out-of-hospital deliveries treated by EMS.	Retrospective and non-randomized.	Oxytocin administered by ambulance personnel after an unplanned out-of-hospital delivery was not associated with diminished PPH.	6

**Table 2:** Comparison of recommendations from different organizations for the treatment of PPH.

Aspect	The Primary Cause of PPH Recognized as	First-Line Uterotonic Drug	Second-Line Uterotonic Agent	Adjunct Therapy	Timing of TXA
WHO	Uterine atony	Oxytocin 10 IU IM/IV. IV infusion of 10–40 IU in 1000 mL isotonic crystalloids flow. Maximum dose not specified.	Ergometrine 0.2 mg IM/IV (if no hypertension and/or preeclampsia). Misoprostol 800 µg sublingual (if oxytocin is unavailable). Carboprost 250 µg IM (if no asthma).	Tranexamic Acid (TXA) 1 g IV within 3 h of birth.	Administer as soon as possible, within 3 h of PPH onset.
FIGO	Uterine atony	Oxytocin 10 IU IM/IV. IV infusion of 10–40 IU in 1000 mL isotonic crystalloids at 100–200 mL/h flow. Maximum dose not specified.	Misoprostol 800 µg sublingual or rectal. Ergometrine 0.2 mg IM/IV (if no hypertension and/or preeclampsia). Carboprost 250 µg IM (if no asthma).	Tranexamic Acid (TXA) 1 g IV in cases of trauma or coagulopathy.	Administer as early as possible.
RCOG	Uterine atony	Oxytocin 5 IU IV slow bolus or 10 IU IM. IV infusion of 40 IU in 500 mL isotonic crystalloids at 125 mL/h flow. The maximum dose for IV infusion is 40 IU.	Ergometrine 0.5 mg slowly IV/IM (if no hypertension and/or preeclampsia). Carboprost 250 µg IM. repeated at intervals of not less than 15 min to a maximum of eight doses (if no asthma). Misoprostol 600 µg orally or 800 µg rectally.	Tranexamic Acid (TXA) 1 g IV, repeat if needed after 30 min.	Administer as soon as possible, within 3 h of onset.
ACOG	Uterine atony	Oxytocin 10–40 IU IV infusion or 10 IU IM. IV infusion of 10–40 IU in 1000 mL isotonic crystalloids. The maximum dose for IV infusion is 40 IU.	Methylergonovine 0.2 mg IM. Carboprost 250 µg IM (if no asthma). Misoprostol 800–1000 µg rectally.	Tranexamic Acid (TXA) 1 g IV, may repeat after 30 min.	Administer within 3 h of PPH onset.
SOGC	Uterine atony	Oxytocin 10 IU IM or 20–40 IU IV infusion. IV infusion of 10–40 IU in 1000 mL isotonic crystalloids at 125 mL/hour flow.	Ergometrine 0.2 mg slowly (over 60 s) IM/IV (if no hypertension and/or preeclampsia). Carboprost 250 µg IM (if no asthma). Misoprostol 600–1000 µg rectally or orally.	Tranexamic Acid (TXA) 1 g IV, repeat after 30 min if needed.	Administer within 3 h of PPH onset.
RANZCOG	Uterine atony	Oxytocin Initial 5 IU IV slow bolus 10 IU IM/IV. IV infusion of 10–40 IU in 1000 mL isotonic crystalloids. The maximum dose for IV infusion is 40 IU.	Misoprostol 800–1000 µg rectally or sublingually. Carboprost 250 µg IM repeated at intervals of not less than 15 min to a maximum of eight doses (if no asthma).	Tranexamic Acid (TXA) 1 g IV, repeat if needed after 30 min.	Administer within 3 h of PPH onset.

## Data Availability

All data on which the review is based are available in public databases with references provided in the section. Detailed methodology for literature selection is described in Section 2.
